# Phylogenetic analysis reveals wide distribution of globin X

**DOI:** 10.1186/1745-6150-6-54

**Published:** 2011-10-17

**Authors:** Jasmin Dröge, Wojciech Makałowski

**Affiliations:** 1 Institute of Bioinformatics, Faculty of Medicine, University of Muenster, Niels Stensen Str. 14, 48149 Muenster, Germany

## Abstract

The vertebrate globin gene repertoire consists of seven members that differ in terms of structure, function and phyletic distribution. While hemoglobin, myoglobin, cytoglobin, and neuroglobin are present in almost all gnathostomes examined so far, other globin genes, like globin X, are much more restricted in their phyletic distribution. Till today, globin X has only been found in teleost fish and *Xenopus*. Here, we report that globin X is also present in the genomes of the sea lamprey, ghost shark and reptiles. Moreover, the identification of orthologs of globin X in crustacean, insects, platyhelminthes, and hemichordates confirms its ancient origin.

## Findings

Globins are small heme proteins that bind various external ligands, such as oxygen and nitric oxide, and they are found in all kingdoms of life [1]. Best known are hemoglobin (Hb) and myoglobin (Mb), which have been studied for a long time to understand relationships between protein function and structure. In the past ten years, several new members of the globin gene family were added to the vertebrate's globin gene repertoire. Neuroglobin (Ngb) and cytoglobin (Cygb) are highly conserved and present in all gnathostomes examined till today [2-4]. Other novel globin genes are much more restricted in their phyletic distributions, e.g. globin Y (GbY) has been found in *Xenopus*, lizards, and platypus while globin E (GbE) is only expressed in the eyes of birds [5-7]. Finally, globin X (GbX) seems to be restricted to the genomes of teleost fish and *Xenopus *[5,8]. The physiological functions of these novel globin proteins are unknown, although a species-specific regulation of GbX under hypoxic conditions has been observed [9,10]. Here, we show that the GbX gene is not just restricted to teleost fish and *Xenopus *genomes, but is also present in the genomes of sea lamprey, ghost shark, and reptiles. Additionally, several invertebrate orthologs of GbX were detected in crustacean, insects, platyhelminthes, and hemichordates, confirming its ancient origin.

The BLAST algorithms [11] were employed to search the non-redundant protein database of NCBI, the sequence databases at the GenBank and the genomic sequences of the sea lamprey, the ghost shark, the Burmese python, and the green anole lizard for homologous globin proteins. Corresponding nucleotide sequences were extracted from the genomes and translation start sites and splice sites were predicted using GENSCAN and GeneNet2, respectively, and manually adjusted [12-14]. The ExPASy Molecular Biology Server at the Swiss Institute of Bioinformatics was used for the translation of the putative CDS (http://www.expasy.org). A table with accession numbers of sequences used in the consecutive analyses and a table with CDS sequences of the newly annotated genes are provided in the supplementary materials [see Additional file 1]. The derived sequences were initially aligned using the MUSCLE program and manually refined [15]. The alignment is provided in the supplementary materials [see Additional file 2]. Phylogenetic trees were reconstructed using maximum likelihood and Bayesian approaches. The program package RAxML 7.2.8 [16,17] was employed for maximum likelihood analyses using the rapid bootstrapping algorithm with 1,000 bootstrap replications. Bayesian analyses were conducted in MrBayes 3.1.2 [18,19] using default priors. Two simultaneous Metropolis-coupled Markov chain Monte Carlo samplings were performed with one cold and three heated chains that were run for 5,000,000 generations. The trees were sampled every 100^th ^generation and the 'burn in' was set to 25%. For the calculation of the phylogenetic trees, the CIPRES portal was used (CIPRES Web Portal V3.1) [20]. Phylogenetic analyses were based on the WAG [21] model of amino acid evolution assuming gamma distribution of substitution rates, as suggested by analysis of the alignment with ProtTest3 [22]. The phylogenetic trees were visualized with iTOL [23].

Similarity searches for putative GbX proteins in the nr protein database of NCBI using GbX of zebrafish as query [GenPept: NP_001012261] resulted in the identification of several homologous invertebrate globin proteins and a partial GbX sequence of the green anole lizard [GenPept: XP_003228427]. A tBLASTn search against several vertebrate genomes revealed that GbX is not restricted to teleost fish and amphibians. The GbX gene is also present in the genomes of green anole lizard, Burmese python, ghost shark, and sea lamprey. While teleost fish possess only one copy of the GbX gene, *X. tropicalis *may indeed contain two GbX genes [5]. For lizard and python we obtained sequences that each correspond to a single GbX gene. Interestingly, ghost shark and sea lamprey may possess two copies of the GbX gene. The coding regions of fish and amphibian GbX are distributed on five exons. The introns are located at positions B12.2 (i.e. between the second and third base of the 12^th ^codon in globin helix B), G7.0, E10.2 and H10.0 [8]. The putative lizard and python GbX genes consist of four coding exons and were found on chrUn_GL343635 and on several contigs (contig24997472, contig25376860, contig25192522, contig25757037), respectively. We couldn't locate the first coding exon of fish and *Xenopus *GbX, comprising the N-terminal extension and some part of the globin domain (on average the first 57 amino acids), completely in the lizard and partially in the python genome. Moreover, in python the last coding exon comprising the C-terminal extension of the protein seems to be absent. Interestingly, we were able to identify the first coding exon in the previous assembly of the lizard genome (anoCar1.0). Thus, it is most likely that complete first and last coding exons couldn't be found due to missing sequence data and/or due to miss-assembly. The potential CDSs of lizard and python GbX translate into peptides of 200 and 129 amino acids, respectively. The putative ghost shark and sea lamprey GbX genes consist of five coding exons and are distributed on several scaffolds (AAVX01210948, AAVX0160156, AAVX01601570, AAVX01646770, AAVX01022872, AAVX01385372, AAVX01173359, AAVX01250480) and contigs (contig19308, contig21159, contig11157, contig27503), respectively. The putative cDNAs of ghost shark and sea lamprey GbX translate into peptides of 193 and 222 amino acids, respectively. Interestingly, in both species we found partial sequences of a putative second GbX gene (GbX-2). The partial GbX-2 genes of lamprey and shark reside on contig20413, contig362 and on AAVX01509327, AAVX01477794, respectively. For the sea lamprey coding exons two to four were identified that translate into a peptide of 103 amino acids that is 71% identical and (based on BLOSUM62) 88% similar to the other copy of GbX (GbX-1). For the ghost shark only coding exons two and three were identified that translate into a peptide of 75 amino acids. The GbX-2 peptide of the ghost shark is 67% identical and 83% similar to GbX-1. Since large parts of the putative GbX-2 genes couldn't be found in the genomes, those sequences might represent pseudo-genes. The key residues important in oxygen binding, like the proximal and distal histidines in the E- and F-helix (HisE7, HisF8) and the phenylalanine in the CD region (PheCD1) are strictly conserved in all new annotated GbX proteins.

**Figure 1:**
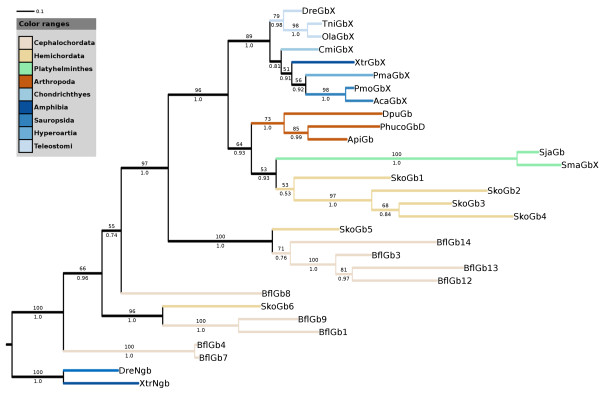
**Maximum likelihood tree of GbX, Ngb and several invertebrate globins**. Maximum likelihood tree of GbX, Ngb and several invertebrate globins. Ngb sequences were included to root the tree. Numbers above branches correspond to bootstrap values, and those below to Bayesian posterior probabilities. Only Bootstrap values above 50% are shown. Branches of vertebrates are shaded in blue. Abbreviations: *Pediculus humanus corporis *GbD (PhucoGbD), *Daphnia pulex *Gb (DpuGb), *Acyrthosiphon pisum *Gb1 (ApiGb1), *Schistosoma mansoni *GbX (SmaGbX), *S. japonicum *Gb (SjaGb), *Saccoglossus kowalevskii *Gb1 (SkoGb1) [GenPept: XP_002739222], *S. kowalevskii *Gb2 (SkoGb2) [GenPept: XP_002739227], *S. kowalevskii *Gb3 (SkoGb3) [GenPept: XP_002739229], *S. kowalevskii *Gb4 (SkoGb4) [GenPept: XP_002739228], *S. kowalevskii *Gb5 (SkoGb5) [GenPept: XP_002733371], *S. kowalevskii *Gb6 (SkoGb6) [GenPept: XP_002732485], Ngb *Xenopus tropicalis *(XtrNgb), Ngb *Danio rerio *(DreNgb), GbX *X. tropicalis *(XtrGbX), GbX *T. nigroviridis *(TniGbX), GbX *D. rerio *(DreGbX), GbX *Oryzias latipes *(OlaGbX), GbX *Callorinchus milii *(CmiGbX), GbX *Python molurus *(PmoGbX), GbX *Anolis carolinensis *(AcaGbX), GbX *Petromyzon marinus *(PmaGbX), *Branchiostoma floridae *Gb1 (BflGb1) [GenPept: XP_002608549], *B. floridae *Gb3 (BflGb3) [GenPept: XP_002610016], *B. floridae *Gb4 (BflGb4) [GenPept: XP_002589215], *B. floridae *Gb7 (BflGb7) [GenPept: CBL51553], *B. floridae *Gb8 (BflGb8) [GenPept: XP_002608525], *B. floridae *Gb9 (BflGb9) [GenPept: XP_002601010], *B. floridae *Gb12 (BflGb12) [GenPept: XP_002605405], *B. floridae *Gb13 (BflGb13) [GenPept: XP_002598546], *B. floridae *Gb14 (BflGb14) [GenPept: XP_002610160].

The newly found vertebrate GbX proteins, the putative orthologous globin proteins of the lancelet [24] as well as several homologous invertebrate globin proteins were added to the alignment of vertebrate GbX and Ngb proteins. We refrained from including the GbX-2 proteins of the ghost shark and sea lamprey because of the incompleteness of the data. The sequences of pea aphid and *Daphnia pulex *globin proteins were corrected based on available EST sequences (GL350495, FF335385, FE368658, FE407975 and FE407974). Various phylogenetic trees were constructed employing the maximum likelihood and Bayesian algorithms. Because of the high divergence of non-metazoan globin proteins and in order to identify orthologous relationships between GbX and invertebrate globin proteins, the closest vertebrate relative of GbX, namely Ngb, was treated as an outgroup (DreNgb, XtrNgb). As expected, the new annotated GbX proteins from sea lamprey, ghost shark and reptiles group with GbX from teleost fish and *Xenopus *with high bootstrap support (Figure 1). However, the clustering of the GbX proteins is not in accordance with the species tree. For example, in our phylogenetic trees GbX from sea lamprey is closer related to GbX of reptiles than to GbX of the ghost shark. The discrepancy of gene and species tree may be caused by the overall comparable similarity of the GbX proteins which ranges from 70% to 88% (based on BLOSUM62). However, under the assumption that the gene tree is correct, we would assume a scenario in which the last common ancestor of vertebrates possessed multiple copies of GbX and in which several losses of GbX genes occurred in the evolution of vertebrates. In this scenario the GbX proteins of e.g. teleost fish and reptiles would rather be paralogs than orthologs. Analyses of additional vertebrate genomes will be necessary to resolve this issue. Interestingly, in all phylogenetic reconstructions, the vertebrate GbX proteins consistently group with putative globin proteins of the pea aphid (ApiGb1), human body louse (PhucoGbD), *Daphnia pulex *(DpuGb), two *Schistosoma *species (SjaGb, SmaGb), and the acorn worm (*Saccoglossus kowalevskii*) (SkoGb1-4). This branching was supported by bootstrap support up to 96% depending on the number of analyzed species and the kind of analysis. Support for orthology of these globin proteins was further obtained by analyzing the organization at the genomic level. Within teleost fish and *Xenopus*, the genomic region containing GbX is conserved in gene order and arrangement. The GbX gene of the zebrafish resides on chromosome 17 between the genes for signal recognition particle 14 (srp14) and pleckstrin homology domain-containing family G, member 3 (plekgh3) [5,24]. The putative orthologous globin proteins of the acorn worm (SkoGb1-4) lie next to each other on one genomic scaffold and thus may have arisen by duplications of an ancestral GbX gene. Interestingly, they are close to the putative ortholog of srp14 [see Additional file 3]. Thus, we propose that these globins are orthologs of vertebrate GbX. Further phylogenetic interpretation of the tree suggests that the monophyletic clade, comprising the putative orthologous globin proteins of the lancelet (BflGb3, BflGb12-14) and an additional putative globin from the acorn worm (SkoGb5), contains paralogs of vertebrate GbX. The SkoGb5 gene of the acorn worm resides in a similar genomic neighborhood as GbX of the analyzed euteleostei fish species. While the direct neighbors of vertebrate GbX are not detectable on this scaffold, the homologs of gremlin 1 (grem1) and dnaj (HSP40) homolog subfamily C member 17 (dnajc17), both located downstream of euteleostei fish GbX, reside in close proximity of SkoGb5. Hence, it is likely that this genomic organization arose by a duplication of the region comprising the ancestral GbX gene. Thus, we propose that the globin SkoGb5 of the acorn worm as well as the amphioxus globins (BflGb3, BflGb12-14) are paralogous to vertebrate GbX and that, most probably, the lancelet and higher vertebrates have each lost one copy of GbX.

Our phylogenetic analysis supports the scenario that the GbX gene is a product of a duplication event that predates the divergence between protostomes and deuterostomes [8,24]. As reported before, we were not able to detect GbX in the genomes of mammals and birds. Thus, it seems that GbX was lost twice in vertebrate evolution. Interestingly, the vertebrate lineages that possess a GbX gene represent ectothermic animals while those who lost the GbX gene are endotherms. This distribution may give some hints towards GbX function. Moreover, it will be interesting to see if the ectothermic crocodiles, which are a sister group of birds, own a GbX gene. Crocodiles possess a four-chambered heart which is a characteristic of endotherms in which higher metabolic rates would select for the perfect separation of blood. This led to the hypothesis that the ancestors of crocodilians and birds were endothermic [25]. It will be intriguing to see if GbX was lost in the lineage leading to birds or if was already lost in stem archosaurs.

## Competing interests

The authors declare that they have no competing interests.

## Authors' contributions

JD designed the study, carried out the bioinformatic analyses and drafted the manuscript. WM conceived of the study, participated in its design and coordination and helped to draft the manuscript. All authors read and approved the final manuscript.

## Reviewers' comments

### Reviewer's report 1: Gáspár Jékely, Max Planck Institute for Developmental Biology, Tübingen, Germany

This paper describes novel globin X genes from several vertebrates and invertebrates. The classification of these genes as globin X is confirmed by molecular phylogeny.

Since some nodes in the phylogeny are not well resolved, and there are some groupings inconsistent with the species tree (e.g. the sequences from the deuterostome Saccoglossus grouping with protostome ones), it would be worth showing the alignments as a separate figure. In the alignment members of other globin families should also be included. This could reveal if the globin X group has some shared sequence signatures, distinguishing them from other globins.

The authors should also comment on the putative amphioxus globin X orthologues described in ref 24. The tree in Figure 1 indicates that the amphioxus genes (BflGb3,12,13,14) represent a distinct class, maybe originating at a separate duplication event, predating bilaterian radiation. BflGb6 is missing from the tree.

#### Author's response

Thank you for the insightful comments. Globin sequences are difficult to align, indeed. We agree that in such cases it is useful to provide the alignment, which is now available in phylip format as an additional file 2. The amphioxus globin BflGb6 was excluded from the analysis because no globin domain was detected using the normal mode of SMART. The sequence may be mis-annotated since a part of the globin domain is absent in the current version. Other members of the vertebrate globin gene family were not added to the alignment to improve readability of the tree. Moreover, globin X sequences share only little sequence similarity with other vertebrate globin types. We agree that the amphioxus genes (BflGb3, 12, 13, 14) represent a distinct class derived from a separate duplication event predating bilaterian radiation.

### Reviewer's report 2: Arcady Mushegian, Stowers Institute for Medical Research, United States of America

This is an interesting observation of broader-then-expected distribution of globin X among metazoa, and of the possible connection between globin X and ectothermy. It is suitable for publication as a Discovery Note, with perhaps one clarification. How do we know what was the ingroup and what was the outgroup, i.e., what are X and what are non-X? Apparently, these calls were made before tree inference, but in principle, the ultimate orthology assignment can be only done by examination of the phylogenetic tree. I suspect that the answer is that globin X sequences were much closer to each other than to any other globins, and therefore the extent of the family could be defined without tree mapping - but this was not stated in the manuscript.

#### Author's response

Thank you for your comment that gives us the opportunity to clarify our methodology. The analysis was conducted without prior expectations. A BLAST search was carried out using vertebrate globin X as query to search for similar sequences in invertebrate species. Subsequently, phylogenetic trees were computed to identify relationships between sequences. Neuroglobin was chosen as an outgroup, because it is assumed that neuroglobin emerged before the split of protostomier and deuterostomier. Moreover, globin X is closer related to neuroglobin than to any other vertebrate globin type with identity values ranging from 26% to 34.6%.

## Supplementary Material

Additional file 1 **Tables of used sequences**. A table of used sequences, along with accession number and gi-number, and a table of CDS sequences of the new annotated sequences are provided in this file. Click here for file

Additional file 2 **Amino acid sequence alignment of GbX from zebrafish, medaka, *Tetraodon *and *Xenopus*, Ngb from zebrafish and *Xenopus*, the newly annotated vertebrate GbX proteins, the putative orthologous globin proteins of the lancelet and several homologous invertebrate globin proteins**. The alignment is provided in interleaved phylip format. Click here for file

Additional file 3 **Schematic comparison of the gene neighborhood of GbX from medaka chromosome 22 to scaffolds 14411 and 38908 of the *S. kowalevskii *genome and to scaffold 29 of the *B. floridae *genome**. Arrows indicate the location of the genes (right handed arrow = plus strand, left handed arrow = minus strand). Genes drawn in the same color are homologs. Genes drawn in light grey are not homologous to other genes in the same chromosomal position of the other species. Dots indicate that shown genes are separated by more than one gene. Click here for file
