# Supplementary material for: Phylogenetic analysis reveals wide distribution of globin X
Source: Biol Direct. 2011 Oct 17;6:54. doi: 10.1186/1745-6150-6-54 (PMC3206486; doi:10.1186/1745-6150-6-54)
Supplement: Additional file 1 — Tables of used sequences. A table of used sequences, along with accession number and gi-number, and a table of CDS sequences of the new annotated sequences are provided in this file. [file 1745-6150-6-54-S1.DOC]

Table 1: List of sequences used in this study, along with accession number and gi-number (* indicates sequences that were manually annotated).

| **organism** | **common name** | **accession number** | **gi-number** | **description** |
| --- | --- | --- | --- | --- |
| *Acyrthosiphon pisum* | pea aphid | NP_001191927.1 | 326368295 | Ngb-like |
| *Anolis carolinensis* | green anole lizard | XP_003228427.1 | 327287420 | GbX* |
| *Branchiostoma floridae* | Florida lancelet | CBL51553.1 | 327360022 | globin |
| *Branchiostoma floridae* | Florida lancelet | XP_002589215.1 | 260788354 | BRAFLDRAFT_74626 |
| *Branchiostoma floridae* | Florida lancelet | XP_002608525.1 | 260827144 | BRAFLDRAFT_92393 |
| *Branchiostoma floridae* | Florida lancelet | XP_002601010.1 | 260812603 | BRAFLDRAFT_96956 |
| *Branchiostoma floridae* | Florida lancelet | XP_002608549.1 | 260827192 | BRAFLDRAFT_98913 |
| *Branchiostoma floridae* | Florida lancelet | XP_002605405.1 | 260820164 | BRAFLDRAFT_74222 |
| *Branchiostoma floridae* | Florida lancelet | XP_002598546.1 | 260807499 | BRAFLDRAFT_66937 |
| *Branchiostoma floridae* | Florida lancelet | XP_002610016.1 | 260830134 | BRAFLDRAFT_99970 |
| *Branchiostoma floridae* | Florida lancelet | XP_002610160.1 | 260830423 | BRAFLDRAFT_77082 |
| *Callorhinchus milii* | ghost shark | - | - | GbX-1* |
| *Callorhinchus milii* | ghost shark | - | - | GbX-2* |
| *Danio rerio* | zebrafish | NP_001012261.2 | 147902629 | GbX |
| *Danio rerio* | zebrafish | NP_571928.1 | 18859087 | Ngb |
| *Daphnia pulex* | common water flea | EFX89883.1 | 321478927 | DAPPUDRAFT_231989 |
| *Oryzias latipes* | Japanese medaka | ENSORLP00000021338 | - | GbX |
| *Pediculus humanus corporis* | human body louse | XP_002427507.1 | 242013633 | globin D |
| *Petromyzon marinus* | sea lamprey | - | - | GbX-1* |
| *Petromyzon marinus* | sea lamprey | - | - | GbX-2* |
| *Python molurus* | Indian python | - | - | GbX* |
| *Saccoglossus kowalevskii* | acorn worm | XP_002733371.1 | 291226786 | Ngb-like |
| *Saccoglossus kowalevskii* | acorn worm | XP_002739222.1 | 291238620 | globin D |
| *Saccoglossus kowalevskii* | acorn worm | XP_002739228.1 | 291238616 | globin D |
| *Saccoglossus kowalevskii* | acorn worm | XP_002739229.1 | 291238618 | GbX-like |
| *Saccoglossus kowalevskii* | acorn worm | XP_002739227.1 | 291238614 | globin D |
| *Saccoglossus kowalevskii* | acorn worm | XP_002732485.1 | 291224991 | Ngb-like |
| *Schistosoma japonicum* | flatworm | AAW24922.1 | 56753437 | GbX |
| *Schistosoma mansoni* | flatworm | XP_002576591.1 | 256080649 | - |
| *Tetraodon nigroviridis* | spotted green pufferfish | CAG25725.1 | 56398459 | GbX |
| *Xenopus (Silurana) tropicalis* | western clawed frog | NP_001011196.1 | 58332516 | GbX |
| *Xenopus (Silurana) tropicalis* | western clawed frog | NP_001025522.1 | 71896523 | Ngb |

Table 2: List of CDS sequences of newly annotated vertebrate GbX genes.

| **organism** | **description** | **CDS sequence** |
| --- | --- | --- |
| *Anolis carolinensis* | GbX | ATGGGTTGCGCTTTGTCTGGCGCACAGGATCCCCCCGTTTCGGAAGAGTGCTCCCCCCTTGACGATGGGTTAGATCTGAATCGGGAAACCACACTGGGAAGCAATGGGAGAACTACAGAGCCCTTCCCGCTTTCAGGGGCACAGAAGGAGCTGATCCGAGGGTCCTGGGAAATCCTGCACAAAGACATTGCAAGAGTGGGGATCATTGTCTTTATCAGGCTCTTTGAGACCCACCCAGAATGCAAGGATGTCTTTTTCCTCTTCCGTGACATCGACGACTTCCAGCAGCTGAAGATGAGCAAAGAGCTGCAGGCTCATGGCCTCCGGGTCATGTCTTTCATTGAGAAGAGTGTGGCACGAATGGACCAGGAACCGAAACTTCATCACTTGGCCTTCGAATTGGGCAGGAGCCATTGCCGGTATAAAGCGCCTCCCAAATATTATGAGTACATTGGGATCCAGTTCATCCAAGCTGCCCAACCCATCTTGAAAGAGGCCTGGACCCCAGAGACCGAGAAGGCCTGGGAGGGCTTGTTCCAATACCTGGCGGCAACTATGCGGAGAGGCTTCTATAAAGAACAGAAGGCAACGGGCAAGAATTAG |
| *Callorhinchus milii* | GbX-1 | ATGGGGTGTGCGATCTCAGGACCGGGGCAATACCCGGCAAGCGGGAGAGAGGACGTTGTCGCGGTGGCCAGTCTGTCCCTGAGCGACAGGCAGACGCAGCTGGTGAAGGAGACGTGGAGGCTGGTGCAGGAGGACATCGCCAAGGTGGGCATCATCATGTTCGTCAGACTGTTCGAGACACACCCCGAGTGCAAGGACGCCTTCTTCCTGTTCCGAGACATCGATGACCTTCAGCAGCTGAGGAAGAGCAAAGGGCTGCGAGCTCACGGTCTGAGAGTGATGTCGTTCATTGAGAAGACAGTGGCCAGGCTGGACCAGGAGGACCGGCTCCAGCAGCTCGNGTTAGAACTCGGAAAGAGTCACTTCCGCTACAGTGCAGCACCCAAGTACTACCCGTATGTCGGGAATGAATTTATTTGTGCAGTGCAGCCCATCCTGAAGGAGAAGTGGACCGCGGAGGTTGAAGAAGCATGGAAGGGCCTCTTTCACTATCTAACCAGTGTGATGAAAAAAGGCTACCAGGATGAGGAGAGGGGCAGCTGCCCCAGGGAGAAGCCAAAGCATGGCCCCAACTCTGTGTAA |
| *Callorhinchus milii* | GbX-2 | GCTGTTTGAAACTCATCCCGAGTGTAAGGAAGTTTTCTTCCGATTCCGTGACATCGAACTCCAACAGCTGAAGACGAGGAAAGAGCTGCAGTCCCACGGTCTGCGTGTGATGTCCTTCATTGAGAACAGTGTGGCCAGACTGGGGCAGGAGGAGAAACTGGAACAACTGATCTTCGACCTGGGACGTAGTCATCAGAGATACAACGTAAACCCCAAATACTATGAG |
| *Petromyzon marinus* | GbX-1 | ATGGGTTGCACCGTCTCGACGGATGAGCGCACAGGAGCGCAGAGCAGCTCGCAGGGACAGTCGCAGGCTTCGCGCAAGCAACAGCAGCCGGAGCAGCAGCGTGCAGGCAGAGAGGGCCACCAGCCGCCTGGGCCACCGCAAGCGCCGTCCGAGAGTCAGAGGCGGCTGGTGCGCGACTCGTGGCTCGCTCTGCAGTGTGACATCGCTCGTGTGGGAGTCATCATGTTCGTGAGGCTGTTCGAAACCCATCCAGAATGCAAGGATGTGTTTTATCAGTTCCGCGATTGCGAGGACCTCCAGAAACTGAAGATGAACAAGCAACTGCAGGCACATGGGCTGAGGGTGATGTCGTTTATCGAGAAAAGCGTGGCTCGGCTGGAGCAGGAGTGCGTGTTGGAGCAGCTGATCGTGGAGATGGGCAGGAAACACTACAAGTACAATGCATCCCCAAAATACTACTCGTTTGTGGGCATCGAGTTCATCGCTACAGTTCAACCCTTCTTGCAGGAGAAGTGGACTAATGAGGTAGAAGATGCCTGGCAGTGTCTCTTCCGCTACATTGCGGCCGTAATGAAAAGGGGCTACCTGGAGGAAGAGGCGGCCAGCAATGGTGTGAACACCGCCAACTACGACCGTGGCCAGGGAAATCATGGGGCCACTGCAATG |
| *Petromyzon marinus* | GbX-2 | GCTGTTCGAGACGCACCCCGAGTGCAAGGATTCCTTCTTCCAGTTCCGCGACATCGAGGATGTGCAGCGGCTTCGGCTGAGCAAACAGCTGCGCTCCCACGGGCTCAGAGTGATGTCCCTCATCGAGAAGACCGTGGCCAGGTTGGATCAAGACGCCGTTCTGGAGCAGCTGATATTCGAGCTGGGCCGCAAGCACTACAAGTACAACGCTCCTCCCAAGTACTACGAGTTCGTGGGGGCTGAGTTCATATCGGCCGTGAAACCTGTGCTGGGAGATCGGTGGACTCAGGACGTGGATGACGCATGGCAG |
| *Python molurus* | GbX | CAGAAGGAGCTGATTCGGGAGTCCTGGAAAATTCTGCATAAGAACATTACCAGAGTTGGAATCATTGTCTTCATTCGACTCTTTGAGACCCACCCTGAGTGTAAAGACGTCTTTTTCCTGTTCCGTGACATAGATGACCTGCAACAGCTGAAGATGAATAAGGAGCTGCAGGCCCATGGCCTCAGAGTGATGTCGTTCATTGAGAAGAGCGTGGCACGGCTGGACCAAGAAGGCAAACTGGAAGTTCTGGCCTTTGAGCTGGGCAGGAGTCACTTCCGGTACAAAGCTCCCCCTAACTACTACGAGTACATTGGCATTCAGTTCATCCAGGCTGTTCAGCCAATTCTCAAAGAAGATTGGACTCTGGAAGTTGAGAAGGCCTGGAAG |
