# Supplementary material for: Phylogenetic analysis reveals wide distribution of globin X
Source: Biol Direct. 2011 Oct 17;6:54. doi: 10.1186/1745-6150-6-54 (PMC3206486; doi:10.1186/1745-6150-6-54)
Supplement: Additional file 2 — Amino acid sequence alignment of GbX from zebrafish, medaka, Tetraodon and Xenopus, Ngb from zebrafish and Xenopus, the newly annotated vertebrate GbX proteins, the putative orthologous globin proteins of the lancelet and several homologous invertebrate globin proteins. The alignment is provided in interleaved phylip format. [file 1745-6150-6-54-S2.DOC]

30 351

AcaGbX ---------- ---------- ---------- ---------- ---------M

ApiGb ---------- ---------- ---------- ---------- ---------M

BflGb1 ---------- ---------- ---------- ---------- ---------M

BflGb12 ---------- ---------- ---------- ---------- ---------M

BflGb13 ---------- ---------- ---------- ---------- ---------M

BflGb14 ---------- ---------- ---------- ---------- ---------M

BflGb3 ---------- ---------- ---------- ---------- ---------M

BflGb4 ---------- ---------- ---------- ---------- ---------M

BflGb7 ---------- ---------- ---------- ---------- ---------M

BflGb8 ---------- ---------- ---------- ---------- ---------M

BflGb9 ---------- ---------- ---------- ---------- ---------M

CmiGbX ---------- ---------- ---------- ---------- ---------M

DpuGb ---------- ---------- ---------- ---------- ---------M

DreGbX ---------- ---------- ---------- ---------- ---------M

DreNgb ---------- ---------- ---------- ---------- ----------

OlaGbX ---------- ---------- ---------- ---------- ---------M

PhucoGbD ---------- ---------- ---------- ---------- ---------M

PmaGbX ---------- ---------- ---------- ---------- ---------M

PmoGbX ---------- ---------- ---------- ---------- ----------

SjaGb ---------- ---------- ---------- ---------- ---------M

SkoGb1 ---------- ---------- ---------- ---------- ---------M

SkoGb2 ---------- ---------- ---------- ---------- ---------M

SkoGb3 ---------- ---------- ---------- ---------- ---------M

SkoGb4 ---------- ---------- ---------- ---------- ---------M

SkoGb5 ---------- ---------- ---------- ---------- ---------M

SkoGb6 ---------- ---------- ---------- ---------- ---------M

SmaGbX MDDQIITVES SQSLDSIILH NSNDKDVKCL DDCVHLNTIK SSSINITYDE

TniGbX ---------- ---------- ---------- ---------- ---------M

XtrGbX ---------- ---------- ---------- ---------- ---------M

XtrNgb ---------- ---------- ---------- ---------- ----------

GCALSGAQDP PVSEECSPLD DGLDLN---- ---------- ----------

GNAGTTRRGS IFSKQDSTGD DGSTRHNKRL SSRQNTFLE- ----------

ATTCMQTNKV GLPQHTSQKG TIAGSLHKQS YRRLDSTSGV ADIYSKDDMG

GSGASRPTPR KRKAKKG--- ---------- ---------- ----------

GCEMSTDGQA LSSVIRKDRS E--------- ---------- ----------

GANMGCSNSK KMSHESESAN SGDS------ ---------- ----------

GCSASMTGMG RAGP------ ---------- ---------- ----------

GTIADGEGTE LNGY------ ---------- ---------- ----------

GTIADGEGTE LNGY------ ---------- ---------- ----------

STDRS----- ---------- ---------- ---------- ----------

G--------- ---------- ---------- ---------- ----------

GCAISGPGQY PASGR----- ---------- ---------- ----------

ASVA------ ---------- ---------- ---------- ----------

GCAISGSGLT ARAPEI---- ---------- ---------- ----------

---------- ---------- ---------- ---------- ----------

GCAISGLAAK TDLAE----- ---------- ---------- ----------

SRDKKRSSLS FRSRGSFDVG SS-------- ---------- ----------

GCTVSTDERT GAQSSSQGQS QASRKQQQPE Q--------- ----------

---------- ---------- ---------- ---------- ----------

T-DLSISPKK LILPPTGNST SRSNSSISVN KSNLTQDVPD DI--------

GCSNSSHNC- ---------- ---------- ---------- ----------

SRFTSR--LS SSTLDNFEAI SNLGWEKKLY EHSSTRTFRT RKRKLTIHNY

GCSNST---- ---------- ---------- ---------- ----------

GNEVA----- ---------- ---------- ---------- ----------

GCTSSAASDR PSKND----- ---------- ---------- ----------

ESTESNTPVK ETTEIN---- ---------- ---------- ----------

GELSTTASLS SSPSLNPTRH ELNTSKPLND SNYSLINSFK KRIKQHLTLS

GCAISSLGAK AEFGD----- ---------- ---------- ----------

GCILSSLGWQ WRDSL----- ---------- ---------- ----------

---------- ---------- ---------- ---------- ----------

---------- ---------- ---------- -----RETTL GSNGRTTEPF

---------- ---------- ---------- -----EEEFP PEPPVLPPPE

AFLTKPFSLV GRLLWKVL-- ---------- ----FSWW-V KQIETPSDVT

---------- ---------- ---------- -----PLPSP QPPKPLDPRL

---------- ---------- ---------- -----LYKSP GIGDREDWRL

---------- ---------- ---------- -----TPPKS STPSALDERL

---------- ---------- ---------- -----ALPEP EAPPPVDPRL

---------- ---------- ---------- --------GG DKEPGGGHGG

---------- ---------- ---------- --------GG EKEPGGGHGG

---------- ---------- ---------- ---------- -------AVV

---------- ---------- ---------- -----SWWGK PVDNTPDDIT

---------- ---------- ---------- ---------- ED-VVAVASL

---------- ---------- ---------- ---------- ----------

---------- ---------- ---------- -----RAGEE ETPA------

---------- ---------- ---------- ---------- ------MEK-

---------- ---------- ---------- -----RSR-- EDAA----VE

---------- ---------- ---------- -----KPDQC SPVLEEQRPP

---------- ---------- ---------- -----QRAAG EGHQPPGPPQ

---------- ---------- ---------- ---------- ----------

---------- ---------- ---------- -----TFIQN EHDQALCNVL

---------- ---------- ---------- -----VSPKK EDSMEQLPPT

TIYPAGLFLL TVYNAMG--- ---------- -----CGSSK INGNVVEEKP

---------- ---------- ---------- ---------- ------ADKP

---------- ---------- ---------- ---------- -KSSRSSTSQ

---------- ---------- ---------- -----PLLDP PPPQELDPRI

---------- ---------- ---------- ---------- -PDDIPDEVT

RNSSLYKITF NSSSSSISST NVNKLNLTQD VPDDITSIQN EHENALNTVT

---------- ---------- ---------- -----RSAEE EDAAAAAAVV

---------- ---------- ---------- --------DH TETSPLLPTL

---------- ---------- ---------- ---------- ------MEKD

PLSGAQKELI RGSWEILHKD IARVGIIVFI RLFETHPECK DVFF-LFRDI

PLTMRQKELL TEMWKLLEED IAKVGVITFV SLFETHPDVQ QSFM-PFKGV

GLTPTQSRLV KESWKMFLSK KRENGFVIFR VLFTDYPVTR KLFK-GVEQL

KLDAKEKFFL EKSWKTVARN EDVAAMAMFI NLFRSSPEIK DKWP-QLRKL

PLDAWQRFYL QKSWKTVARK SDQAARTVFL RMLQDNPGLR QKWP-RISLL

PLTQKQKFLL LKSWKGVARQ ISQCGKTMLI RLFKDDPQLM AVFNQKFRHL

PLDARQKFHL EKSWKSVARN IDRAGMFMFL RLFRDCPEMI EKYP-ELRGM

PLTQEQVHGI TETWAILAQD PVERGVDLFM KIFEEDPDLK KLF-YFADDG

PLTQEQVHGI KETWAILAQD PVERGVGLFM KIFEEDPDLK KLF-YFADDG

SLTEGEKATI RRTWAVASRD MMGNGANILL KMFEINPDTK KVFA-KFRNI

GLTANQIQLI RDTWQIVYKN KRENCFAIFR ILFTDHPSTK SLFR-LMDAV

SLSDRQTQLV KETWRLVQED IAKVGIIMFV RLFETHPECK DAFF-LFRDI

ELTDLQKTLL QESWKRLEKD IAQVGIIVFI NLFETHPDMQ SVFL-PFTGV

GLTANHIRLI KESWRLIQED IAKVGIIMFV RLFETHPECK DVFF-LFRDV

-LSEKDKGLI RDSWESLGKN KVPHGIVLFT RLFELDPALL TLFSYSTNCG

HPNEEQIQMI KDSWKVIRDD IAKVGIIMFV RLFETHPECK DVFF-LFRDV

ELTTREKELL IETWKELEEN IAKVGVITFV SLFETHPDVQ ESFM-SFSGV

APSESQRRLV RDSWLALQCD IARVGVIMFV RLFETHPECK DVF-YQFRDC

-----QKELI RESWKILHKN ITRVGIIVFI RLFETHPECK DVFF-LFRDI

SINDEQLLLL QSSWSIVKQH IEKIGVITFL GIFEQHSDFR DAFT-EFRKR

SLTDQHRVIL LDSWKVIQED IAKVGVIMFM GLFETHPECK EVFM-PFKEL

ELTKEQKDTL IQTWQNLHAD LERIGMLMFM GLFEHNPEIK EFFV-GADSR

KLTVEQKRLI IDSWKELHID LERIGMLMFM GMFGTHPQTR E-FF-NFRGT

SLSKEQEKIL VQTWLSIRGD LERIGLLMFT GLFEHHPEAK -VMF-GLSDT

PLTARQKFSI QKSWKAIQRN MEGVGMDIFI RLFKAHPEYQ DLFP-EFKGM

ILTPKEVKAI SESWKVVYAK KKENGVALFI RLFQSVPGSK SLFK-NLDGI

SFTNEQLLLL QTSWSIVKQH IEKIGVITFL GIFEQHSDFR DAFT-EFRKR

YPREDQIQMI KDSWKVIRDD IAKVGIIMFV RLFETHPECK DVFF-LFRDV

NLSEQQQQLL VESWRLIQHD IAKVGVILFV RLFETHPECK DVFF-LFRDV

QLSGPQKELI RESWQTVSQD QLHHGTVLFS RLFELEPELV FLFQYNSSHF

DD--FQQLKM SKELQAHGLR VMSFIEKSVA RM-DQE-PKL HHLAFELGRS

D---LEDLKH SRQLRDHALR VMAFVQKAVA RL-YEP-DKL ETLLRDLGKK

DLDAPGQLES SITLRAHVTR FMHSFDTYME SL-DDP-EDL KQLLYDTGKS

SE---DEMRD SPYLQKLSVR ILGAMDHVID SL-DDP-DYL IPALEKLGQM

TE---EEIPT SPYIKFLGER IFDCLDYIID NL-GDL-DHV ISELTKLGRQ

RERDADVLYQ DAILDAHAAT VMEALHEAIT HL-DDS-VFV MKVLHDVGKM

DD--QEELRN SQFLQEHSQR VLDAFDHTID SL-DDV-DYV IQLLKKIGQM

R----ELSRE DQRMRSHGER VMEAVGAAVD SL-GDL-TAV VPVLTELGAL

R----ELSRD DQRVRSHGER VMEAVGGAVD SQ-GDL-TAV VPVLTELGAL

PD---DQLRS TPRFRAHVTR VMASISTVVN SL-DDQ-EVL LDLFKDIGKK

DLDVPGEFEK NVAARAHMVR FMHSFATFMD TL-DEP-AEL RQLLYDLGKN

DD--LQQLRK SKGLRAHGLR VMSFIEKTVA RL-DQE-DRL QQLXLELGKS

V---LDDLKK SKLLSEHALR VMGAVQRAVH RL-QEP-EKL HAFLSELGRK

ED--LERLRT SRELRAHGLR VMSFIEKSVA RL-DQL-ERL ETLALELGKS

D--APECL-S SPEFLEHVTK VMLVIDAAVS HL-DDL-HTL EDFLLNLGRK

ED--LERLRT NRELRAHGLR VMSFIEKSVA RL-DQP-ERL EALAVELGKS

D---IEDLKH SKQLRAHALR VMAFVQKAVA RL-HEP-EKL ETLLKELGRK

ED--LQKLKM NKQLQAHGLR VMSFIEKSVA RL-EQE-CVL EQLIVEMGRK

DD--LQQLKM NKELQAHGLR VMSFIEKSVA RL-DQE-GKL EVLAFELGRS

K---FVDVKH DPAMQVHGLR VLSIVDKMIT RL-PKT-DDI ELKLMTIGSK

QG---DDLRW SSALKAHGLR VMAVIERVLA RI-DSD-EKI EEHLKALAKK

DMK-TEELRY NEKLQEHGIR VMGLVEKIIS SM-GFEDEKI DQMVVDLGKR

S----DDPKN TQRLREHGLR FMSLVKKILV FI-DEK-PRL DAMLLDLGRR

AMS-PKDKEN TALIKEHGLR FMNVVRDVLT LISEKNGSQA ECVLIDLGRR

SE---EKLRN SINFETHVGI FMNVIDECID SL-EDA-DHV INLLTKKGRK

DDE--EKLRN HPRLKAHGFR VMSSVNSLIE SL-EEG-ELL VQLLKDLGSS

K---FVDIKH DPAMQVHGLR VLSVVDKLIT RL-PKT-DDI EKQLMMIGSK

ED--LERLRS SRELRAHGLR VMSFIEKSVA RL-DQQ-DRL EALAVELGKS

DD--LQALRA NKDLRAHGLR VLSFVEKSVA RI-ADC-ARL EELALELGRS

S-KVQDCL-S SAEFTEHIRK VMTVIDAAVS SL-DCL-SSL DEYLTSLGRK

HCRYKA-PPK YYEYIGIQFI QAAQPILKEA --WTPETEKA WEGLFQYLAA

HYHYGA-KQK YVDLIGPQFI MAIQPSLVDR --WTEEMHSA WTALFLNMAY

HLIHDI-KPE YFDVLETVLM KSLRIVFGSK --LTPQLEEA WQTAYSHLKV

HADMTN--PI ILP------- ---------- -------EDL WVNKA-FLRQ

HSDMNVMTPE DVWAIEAAFL AGVQECLEDR --FTIKYEEI YSRFIVFVIE

HQRYNV-DPS VFLKVEKPFL TAVSEVLGDR --YTKNMEEI YTITIKFILA

HADLEL-KPD DMWKLEQPFL AAVAECLEDR --YTPKFQEI YSKLITFIIE

HHKYGV-QPS YFDTVGAALI YILETNLGDK --LTPSIRQG WVLVYAIVGA

HHKYGV-QPS YFDTVGAALI YILETNLGDK --LTPSIRQG WVLVYGIVGA

HYPARV-PTE YFDVIAGAIL CMLQRCLGTG --YTAEVDSA WTKLYGSLGR

HAKHQV-GPE LFDALGPILM KALPIVLDGK --FTPEVKTA WLTAYTFMST

HFRYSA-APK YYPYVGNEFI CAVQPILKEK --WTAEVEEA WKGLFHYLTS

HEKNGA-KLE YIDYIGPQFL CAIRPILGDD --WTLETEKA WTLLLDYMTA

HYRYNA-PPK YYGYVGAEFI CAVRPILKDR --WTPELEEA WKTLFQYVTS

HQAVGV-NTQ SFALVGESLL YMLQSSLGPA --YTTSLRQA WLTMYSIVVS

HYHYNA-PPK YYNYVGAEFI CAVQPILKEQ --WTTELEKA WQTLFQFVTA

HVGYGA-KQK YVELVGPQFI LAIKPSLEKQ --WDEELDDA WTHLFKIIEF

HYKYNA-SPK YYSFVGIEFI ATVQPFLQEK --WTNEVEDA WQCLFRYIAA

HFRYKA-PPN YYEYIGIQFI QAVQPILKED --WTLEVEKA WK--------

HCRYVP-TIG LISSVSDQLW GAIEPVLKEE GSWSDELAVT WKTVLDYLTK

HVEYGA-NSD LVRLFGPQFI GSMKRQLHKS --WSDEMQDA WTVLFDIIIY

HLGYDV-HIP FIDLFGRQFV FAIKPTLHTH --WTANVEEA WTQLFKYIGY

HQEYKA-DFN LIDVFGEQFI LSVRPTLKHS --WNPDVESA WAQLFKYISY

HCSYNA-DIN LIDVFGQQFI ASIQPTLTGS --WDKKVEDA WIQLFKYIAF

HANYGV-KPE FISDIEEPFL ASVKQLLEDR --YSEKIEEI YKLTIKFILE

HSKNKV-TSS HFDALGPVII WLLQKENGDS --FTPAVKNA WLKGWGVMKS

HCRYVP-TIA LVSSVSDQLW GAIEPVLKEE GLWSADLAVT WKSILDYLTR

HYHYNA-PPK YYSYVGAEFI CAVQPILKER --FTSELEEA WKTLFQYVTG

XYRYNA-PPR YYQYVGTEFI SAVCPMLHDK --WTAEVEEA WKGLFAYICT

HRAVGV-KLE SFNTVGESLL FALESCLGDA --FTSDTREA WSLLYANVVQ

TMRRGFYKE- ---QKATGKN ---------- ---------- ----------

IMKGSMAAEE -RFKVKKTAT ---------- ---------- ----------

TIKQGLEDAI QKRDQADTSV VVTVE----- ---------- ----------

Q--------- ---------- ---------- ---------- ----------

TMVIGFDPH- ---------- ---------- ---------- ----------

TLSEGATMEL TEDEQKNLGR LWRPPGRVHK FVRPEKVAAI VDAQSEENGV

HVVNGFDPH- ---------- ---------- ---------- ----------

TMKKGMQQAM DHQNMAKTRP ---------- ---------- ----------

TMKKGMQQAM DHQNMAKTRP ---------- ---------- ----------

HAKDGLREAA AMGTP----- ---------- ---------- ----------

HLKEGVEEGQ RQLADSK--- ---------- ---------- ----------

VMKKGYQDE- E--RGSCPRE KPKHGPN--- SV-------- ----------

TMKESLVEAR NASAAESSKP LTLPPSSSSS SSAATDD--- ----------

IMREGFLEEE -RNKRSNTQT SSRERPDKRS TAI------- ----------

AMTRGWAKNG EHKSN----- ---------- ---------- ----------

LMKQGYQEE- SARQRQLA-T SPKDRLDKRN TAL------- ----------

VMVSSMDDDR KDQRTLERYI IYI------- ---------- ----------

VMKRGYLEEE AASNGVNTAN YDRGQGNHGA TAM------- ----------

---------- ---------- ---------- ---------- ----------

TVRYGLAKTF HSTHR----- ---------- ---------- ----------

HMTTNMVPEQ PENNNIAKRQ KSSRKSRTKY MIDNGHSQ-- ----------

LMRYGYHTKL QQVQKKNS-- ---------- ---------- ----------

MMKKGMMQTD KNK------- ---------- ---------- ----------

TMKQGLAAEL IDKSLKLNGK P--------- ---------- ----------

HFINGLKESV G--------- ---------- ---------- ----------

VIVGSLEEAY AKMKT----- ---------- ---------- ----------

TXXXXNANLL T--------- ---------- ---------- ----------

LMRKGHQEE- GSRQRHLA-L PPKDGPEKRT SAL------- ----------

VMERGYQEEE -RRHSDGRSL IDGLQGNKGL I--------- ----------

SMSRGWHRDS QEQREGI--- ---------- ---------- ----------

-

-

-

-

-

H

-

-

-

-

-

-

-

-

-

-

-

-

-

-

-

-

-

-

-

-

-

-

-

-
