# Supplementary figures and images for: Phylogenetic analysis reveals wide distribution of globin X
Source: Biol Direct. 2011 Oct 17;6:54. doi: 10.1186/1745-6150-6-54 (PMC3206486; doi:10.1186/1745-6150-6-54)

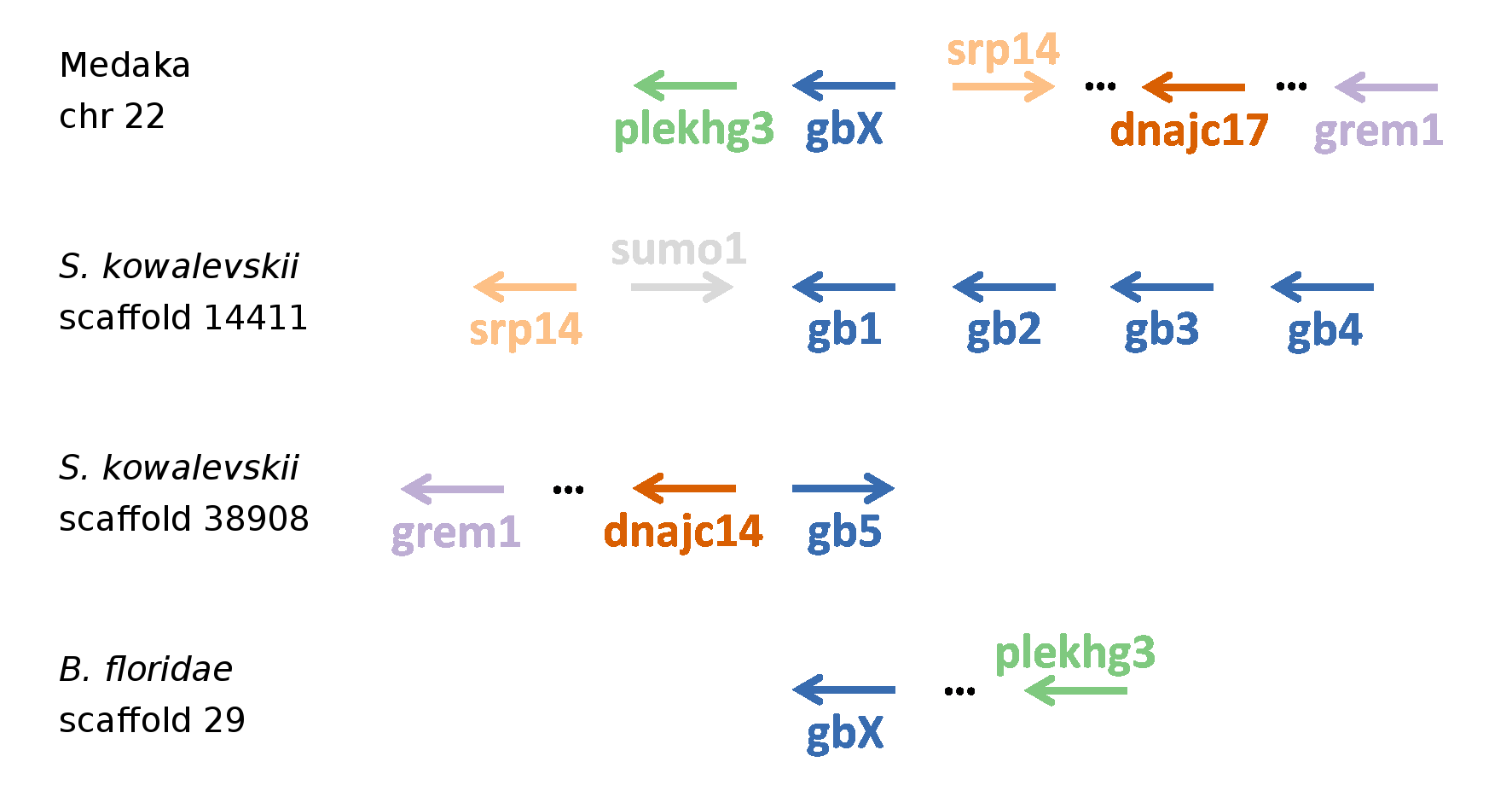

Supplement: Additional file 3 — Schematic comparison of the gene neighborhood of GbX from medaka chromosome 22 to scaffolds 14411 and 38908 of the S. kowalevskii genome and to scaffold 29 of the B. floridae genome. Arrows indicate the location of the genes (right handed arrow = plus strand, left handed arrow = minus strand). Genes drawn in the same color are homologs. Genes drawn in light grey are not homologous to other genes in the same chromosomal position of the other species. Dots indicate that shown genes are separated by more than one gene. [file 1745-6150-6-54-S3.DOC]
